# Chronosequence and direct observation approaches reveal complementary community dynamics in a novel ecosystem

**DOI:** 10.1371/journal.pone.0207047

**Published:** 2019-03-18

**Authors:** Andrew Kulmatiski, Karen H. Beard

**Affiliations:** Department of Wildland Resources and the Ecology Center, Utah State University, Logan, UT, United States of America; US Geological Survey, UNITED STATES

## Abstract

Non-native, early-successional plants have been observed to maintain dominance for decades, particularly in semi-arid systems. Here, two approaches were used to detect potentially slow successional patterns in an invaded semi-arid system: chronosequence and direct observation. Plant communities in 25 shrub-steppe sites that represented a 50-year chronosequence of agricultural abandonment were monitored for 13 years. Each site contained a field abandoned from agriculture (ex-arable) and an adjacent never-tilled field. Ex-arable fields were dominated by short-lived, non-native plants. These ‘weedy’ communities had lower species richness, diversity and ground cover, and greater annual and forb cover than communities in never-tilled fields. Never-tilled fields were dominated by long-lived native plants. Across the chronosequence, plant community composition remained unchanged in both ex-arable and never-tilled fields. In contrast, 13 years of direct observation detected directional changes in plant community composition within each field type. Despite within-community changes in both field types during direct observation, there was little evidence that native plants were invading ex-arable fields or that non-native plants were invading never-tilled fields. The more-controlled, direct observation approach was more sensitive to changes in community composition, but the chronosequence approach suggested that these changes are unlikely to manifest over longer time periods, at least in part because of disturbances in the system. Results highlight the long-term consequences of soil disturbance and the difficulty of restoring native perennials in disturbed semi-arid systems.

## Introduction

Over the past century, early-successional, non-native plants have invaded disturbed areas around the world [1, 2]. Traditional ecological theory suggests that without continued disturbances late-successional species will gradually replace these invaders [3–5]. Yet, in some, particularly semi-arid areas, early-successional, non-native species persist for decades or longer as potentially alternative-state communities [6–10]. Nonequilibrium ecology and resilience theory suggest that disturbances can induce a shift to community types that vary over time but resist returning to a pre-disturbance condition [11, 12]. Distinguishing whether early-successional, non-natives follow successional patterns or if they create stable alternative-state plant communities has important theoretical and management implications [13–16]. Where native perennial species re-establish in years or decades, management efforts may delay restoration [16]. Where alternative-state communities develop or where succession occurs at very slow rates (i.e., centuries), intensive management approaches are likely needed [12–13].

Assessments of long-term patterns of plant community composition are important for understanding how plant communities respond to disturbance and determining appropriate management approaches [17–20]. The data needed to assess long-term community dynamics in semi-arid systems, however, is often lacking for any given site [8, 9, 21–22]. Space-for-time substitutions (or chronosequences) can be used to infer species replacements over long periods. Chronosequence data, however, are susceptible to hidden temporal or spatial variations in factors such as climate, grazing, priority effects, and soil type [10, 23–24]. Direct long-term observations of plant community dynamics can control for these extrinsic factors and provide a better test of community resistance and resilience to changes in species composition but are more difficult to collect [19, 25–26].

Kulmatiski [27] used a chronosequence approach to describe plant community dynamics in a shrub-steppe ecosystem in Washington, USA. Results suggested that non-native and native plants established alternative-state communities in ex-arable fields and never-tilled fields, respectively. It was suggested that disturbance associated with agriculture allowed early-successional, non-native plants to establish, and that once established, these species changed soil conditions in ways that allowed their own persistence (i.e., positive plant-soil feedbacks [27]). To provide a more controlled test of how vegetation dynamics change over time, in the present study the same fields in that chronosequence were monitored for 13 years of direct observation [28]. Direct observation allows an assessment of how the plant community in each individual field changes over time. Direct observation also allows an assessment of the effects of several extrinsic factors that were not possible to address during the previous study. During the course of this study, roughly half of the ex-arable fields were managed (i.e., tilled, herbicided and seeded with restoration seed mixes) to increase native plant growth, most of the fields were burned in a wildfire in the penultimate year of surveying, and across all sites a biocontrol agent nearly eliminated the dominant non-native plant, *Centaurea diffusa* Lam.

We had three objectives in this study. First, we describe community differences in ex-arable and never-tilled fields using 13 years of direction observation data. Second, we use direct observation and chronosequence approaches to determine how the communities in the two fields are changing over time, and if they are changing, at what rate. Differences in the results between the two approaches are discussed. Third, we determine whether management and natural treatments change the trajectories of these communities.

## Methods

### Study area

Research was conducted in the Methow Valley, Washington (WA), USA (48° 37’ N, 120° 10’ W). Precipitation is seasonal with 250 of 360 mm of annual precipitation falling mostly as snow between October and March (http://www.ncdc.noaa.gov). The growing season begins with snowmelt in April and continues until snowfall in November though most native grasses and forbs become dormant by July. Native shrub-steppe communities, dominated by *Purshia tridentata*, *Pseudoroegneria spicata*, *Lupinus sericeus*, *Artemesia tridentata* var. tridentata, and *Balsamorhizae sagittata*, occupy most of the hilly landscape, whereas valley bottoms and benches are largely used for agriculture and, once abandoned, are occupied by various non-native species, including *Centaurea diffusa*, *Medicago sativa*, *Bromus tectorum*, *Poa bulbosa*, and *Cardaria draba*. Unless otherwise noted species naming follows that of Hitchcock and Cronquist [29]. Cattle grazing is limited in the study area but mule deer grazing is common and likely to suppress woody plant growth [30]. Permission to work at the study site was provided by the local land manager for the Washington Department of Fish and Wildlife.

Aerial photographs were used to identify 25 study sites with fields that had been abandoned from dryland agriculture (*Triticum aestivum* and *Medicago sativa*) between 1950 and 1999 and that had adjacent undisturbed fields with similar slope, aspect and soils (S1 Table). Henceforth, these field types are referred to as ‘ex-arable’ and ‘never-tilled’, respectively. At least 200 m but not more than 25 km separated the 25 sites (elevation range: 630 to 1000 m). All sites were located on the Newbon-Concunully association (coarse-loamy, mixed mesic Typic Haploxerolls)[31].

### Vegetation sampling

From 5 to 25 June from 2002 to 2015, plant cover by species was determined in 1 m × 1 m quadrats in each of 25 sites. Each of the 25 sites contained an ex-arable field and a paired never-tilled field. In each field, two transects were established. These transects were parallel to and 5 m or 50 m from the historical tillage boundary. Depending on field length, 15 quadrats were placed at an interval of one every 5 to 10 m in each transect. This sampling design (15 quadrats × 2 transect distances × 2 field types × 25 sites) produced 1,500 quadrats per year. Data was not available for the 2010 season, so 13 years of data are reported. The total dataset contains species compositions for 18,692 quadrats.

Plant cover was measured as percent ground cover using visual estimation by the same observer over the 13 years to the nearest 1% of cover. A 20 cm × 20 cm grid in the quadrat helped guide estimation. Due to the number of plots (1,500 each year) and the request of land managers, transects and plots were not permanently marked so that while fields and transects were resampled each year, specific plots were not resampled each year. Visual estimates of percent cover were well correlated with point-intercept-derived estimates of plant cover by species (i.e., R^2^ = 0.95 to 0.98)[27].

### Management and wildfire history

In 2003 and 2004, the biocontrol agent *Larinus minutus* (Gyllenahal) was released to control the dominant non-native, *C*. *diffusa*. Between 2005 and 2013, 12 of the ex-arable fields were managed to increase native plant abundance (S1 Table). Management included broad-spectrum herbicide application in the spring followed by two to three passes with a disk harrow at two to four different times over two growing seasons (*e*.*g*., spring and fall) prior to a native plant seeding, typically in the fall. In the penultimate year of the study (August 2014), most (15 of the 25) of the sites burned in a wildfire (S1 Table).

### Statistical analyses

To address our first objective, to test for differences between the two field types, we used non-metric multidimensional scaling analyses (NMS)[32]. NMS analyses were conducted on functional group (native annual, non-native annual, native forb, non-native forb, native grass, non-native grass, native perennial, non-native perennial, native shrub) and species matrices using Bray-Curtis dissimilarity matrices. Analysis of similarities (ANOSIM) was used to test for differences in NMS scores between treatments (i.e., ex-arable or never-tilled field)[32–33].

To further address the first objective, we provide a more detailed analysis of differences in community composition between ex-arable fields and never-tilled fields, and between distance transects (5 and 50 m), using generalized linear mixed models (GLMM) with a two-way factorial (field type by distance) in a split-plot design with whole plots (fields) in blocks (sites) and the following response variables: native, non-native, grass, forb, annual and perennial.

To address the second objective, to assess community composition change over time using both direct observation and chronosequence data, linear trend in the NMS1 scores over years-since-abandonment and comparison of trends were assessed using a linear mixed model. In addition to NMS1 scores, this analysis was done on functional groups and species (five dominant in each field type). Fixed effects factors were (1) field type (ex-arable or never-tilled); (2) years-since-abandonment; (3) mean number of years-since-abandonment; (4) interaction of field type with years-since-abandonment, which allowed the estimate of the within-field slope of the regression of NMS1 scores on number of years-since-abandonment to differ for ex-arable and never-tilled fields; and (5) interaction of field type with mean number of years-since-abandonment, which allowed the estimate of the between-field slope of the regression to differ for ex-arable and never-tilled fields [34]. Random effects factors were study site and field-within-study-site, which allowed for random intercepts for fields in the regressions of NMS1 scores on the number of years-since-abandonment. Covariance among the 13 annual repeated measures on a field was modeled using a first-order autoregressive structure. We also fitted a model that allowed for random slopes among field regressions; there was no statistical support for random slopes so we present results for the model with only random intercepts. Number of years-since-abandonment was within-field centered prior to analysis. For simplicity, only data from 50 m transects were analyzed for this analysis.

To test for management effects in ex-arable fields, the third objective, regressions of NMS scores were conducted for managed and unmanaged fields separately. To test for biocontrol effects on the target non-native plant, *C*. *diffusa*, a one-way GLMM with year as the factor and field as the random variable was used to test for differences in cover in each community. To test for the effects of wildfire, differences in NMS values in 2015 were compared to average NMS values from 2002 to 2014, using a t-test for each field type separately.

All multivariate analyses were performed in R using the LabDSV library [35–36]. Regressions and GLMMs were conducted using the Reg and GLIMMIX procedures in SAS v.9.4 TS1M4 for Windows (SAS Institute, Cary, North Carolina, USA).

## Results

### Community composition in ex-arable and never-tilled fields

**Fig 1:**
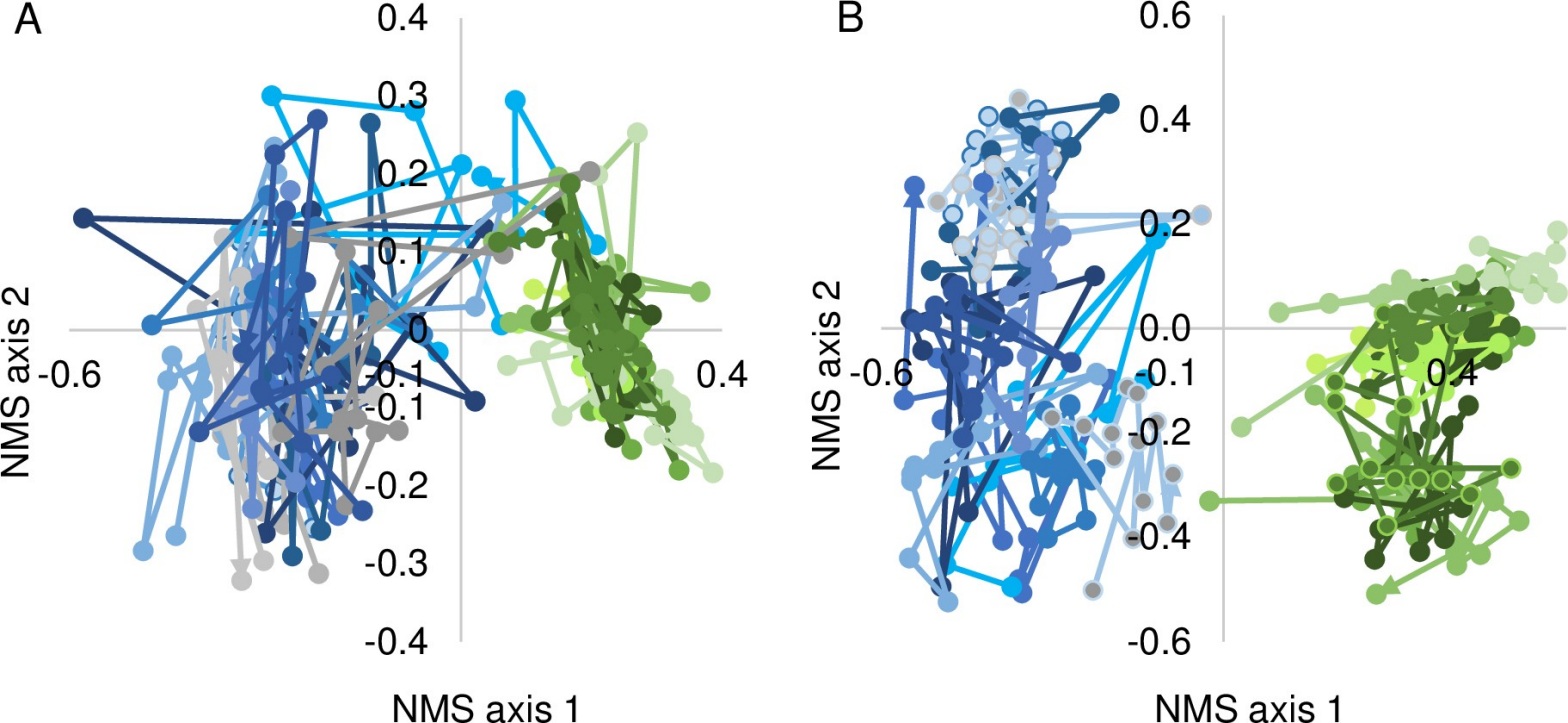
Non-metric multidimensional scaling (NMS) graph by (A) functional group and (B) species of plant community composition in adjacent ex-arable fields (blue and grey colors) and never-tilled fields (green colors) over 13 years. Each point represents the mean composition of vegetation across a transect located 50 m from a tillage boundary. Lines connect values from a field over 13 years of direct observation. For clarity, data from 12 of 25 randomly selected sites are shown.

NMS of the direct observation data revealed differences in functional group and species composition between ex-arable fields and never-tilled fields (ANOSIM statistic = 0.486, 0.444, *P* < 0.001, *P* < 0.001 for functional group and species matrices, respectively, Fig 1). NMS axis 1 scores distinguished differences in plant community composition between ex-arable fields and never-tilled fields, while NMS axis 2 scores distinguished differences in plant community composition among fields and the 13 years of observation (Fig 1).

**Fig 2:**
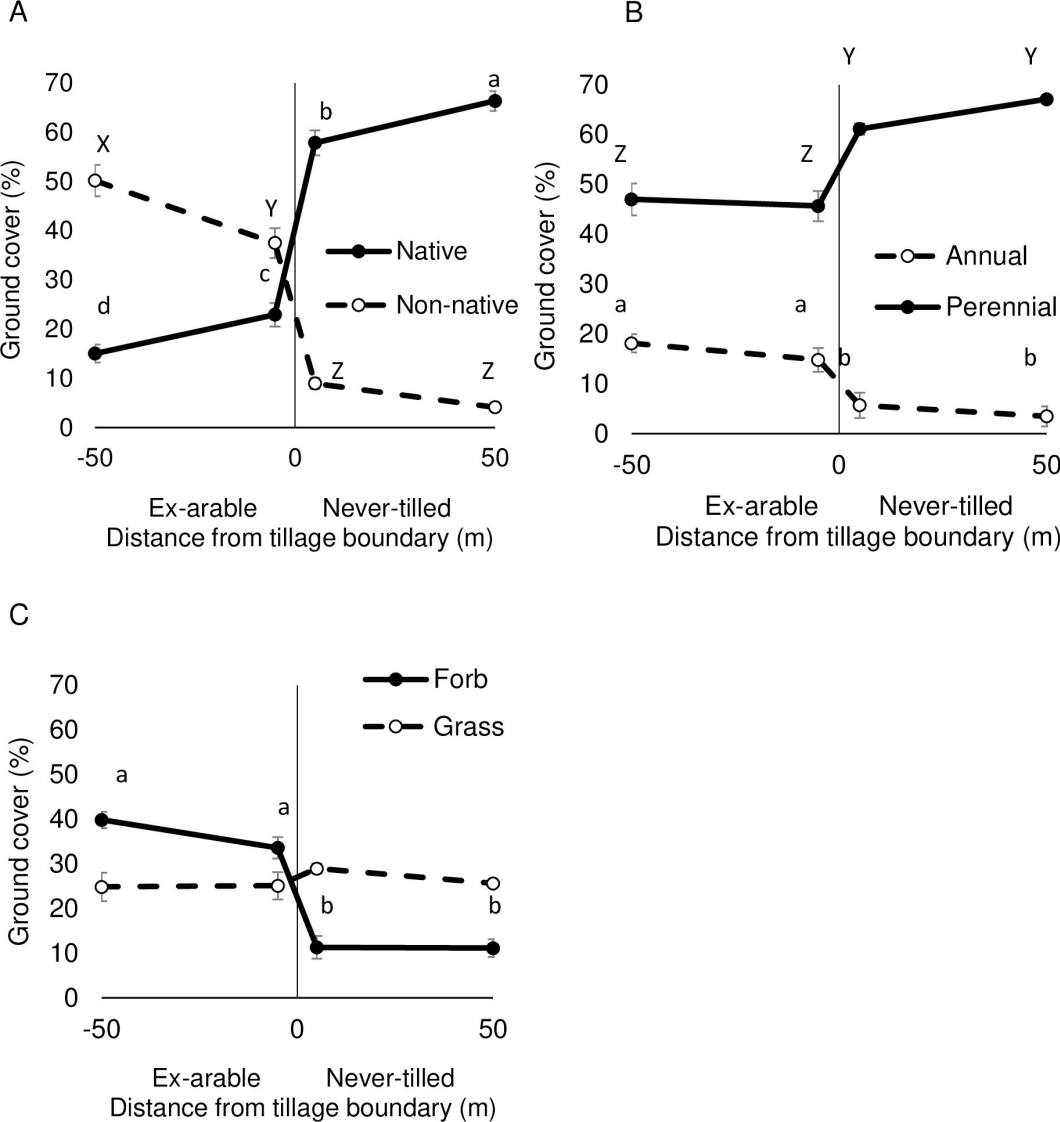
Percent ground cover of (A) native and non-native plants, (B) annual and perennial plants, and (C) forbs and grasses across tillage boundaries. Negative x-axis values are in ex-arable fields. Positive x-axis values are in never-tilled fields. Values represent the mean and standard error associated with the 25 sampled fields (values from replicate plots and years were averaged prior to calculations). Different letters are different at the α = 0.05 level.

Differences in plant functional group composition between ex-arable and never-tilled fields helped explain community-level differences indicated by NMS (Fig 2; Table 1). Non-natives, annuals and forbs were more abundant in ex-arable than never-tilled fields (Fig 2; Table 1). There was no difference in grass abundance between ex-arable and never-tilled fields.

**Table 1:** Mean cover of dominant plant species and functional groups and several community traits in adjacent ex-arable and never-tilled fields, Methow Valley, Washington, USA. Cover values within fields (replicate plots) and among years were averaged prior to calculations so that values represent averages and standard errors associated with the 25 fields.

Measure	Ex-arable fields	Never-tilled fields
Species richness	5.4 ± 0.3 b^1^^,^^2^	6.6 ± 0.3 a
Ground Cover (%)	59.2 ± 1.2 b	68.6 ± 1.6 a
Native:	19.2 ± 1.8 b	62.4 ± 2.1 a
*Pseudoroegneria spicata*	2.8 ± 1.2 b	18.9 ± 1.3 a
*Balsamorhiza sagittata*	0.4 ± 0.1 b	15.3 ± 1.9 a
*Purshia tridentata*	0.1 ± 0.0 b	4.8 ± 0.9 a
*Lupinus* spp.	2.3 ± 0.5 b	4.3 ± 0.5 a
*Artemisia tridentata*	0.1 ± 0.0 b	2.4 ± 0.8 a
Non-native:	39.6 ± 2.1 a	6.6 ± 0.8 b
*Medicago sativa*	7.1 ± 1.7 a	0.3 ± 0.1 b
*Poa bulbosa*	5.9 ± 0.9 a	1.7 ± 0.3 b
*Centaurea diffusa*	5.1 ± 0.8 a	0.2 ± 0.1 b
*Bromus tectorum*	4.5 ± 0.7 a	1.4 ± 0.3 b
*Cardaria draba*	4.3 ± 0.1 a	0.1 ± 0.1 b
Native annual	4.4 ± 0.7 b	2.0 ± 0.2 a
Native forb	7.9 ± 1.0 a	8.2 ± 0.6 a
Native grass	9.8 ± 1.5 b	24.0 ± 0.6 a
Native perennial	14.7 ± 1.7 b	60.4 ± 2.1 a
Native shrub	1.2 ± 0.2 b	30.2 ± 2.7 a
Non-native annual	11.3 ± 1.6 a	2.6 ± 0.5 b
Non-native forb	23.8 ± 2.2 a	3.1 ± 0.4 b
Non-native grass	15.8 ± 1.6 a	3.5 ± 0.5 b
Non-native perennial	28.3 ± 2.4 a	4.0 ± 0.5 b
Non-native shrub	0.0 ± 0.0 a	0.0 ± 0.0 a
Annual	15.8 ± 1.8 a	4.6 ± 0.6 b
Forb	31.7 ± 2.3 a	11.3 ± 0.8 b
Grass	25.7 ± 1.8 a	27.4 ± 1.3 a
Perennial	42.9 ± 1.8 b	64.4 ± 1.9 a
Shrub	1.2 ± 0.2 b	30.2 ± 2.7 a

^1^ Values represent the average from thirty quadrats and 13 sampling years in each plant community in 25 fields (n = 25). The SE represents the error associated with the 25 fields and not error associated with quadrats, transects, or years.

^2^ Values in the same row followed by the same letter were not significantly different at the 0.05 level.

### Community composition across time

**Fig 3:**
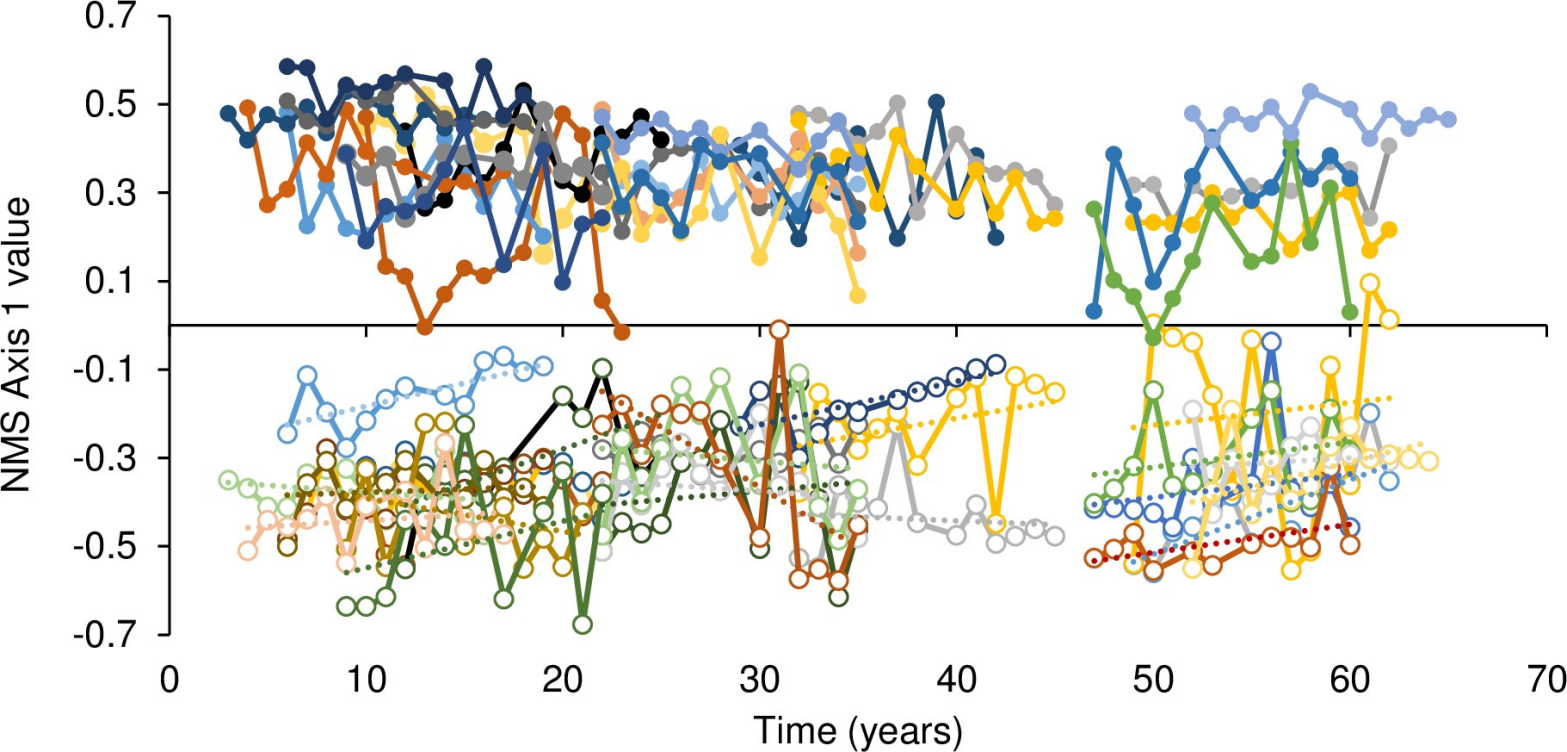
NMS axis 1 scores (species matrix) over time. Data from ex-arable fields shown with open symbols and data from never-tilled fields shown with filled symbols. Data from transects located 50 m from tillage boundaries. Positive y-axis values were associated with native, long-lived perennial communities (Table 1; Fig 1). NMS 1 values increased with time during 13 years of observation in ex-arable fields, but did not change with time in never-tilled communities or across the chronosequence in either community type.

There was evidence for an effect of field type (F_1,22_ = 44.44, *P <* 0.001) and effect of time during direct observation on plant community composition (F_1,78_ = 4.88, *P* = 0.030; Fig 3). There was an interaction between time during direct observation and field type (F_1,78_ = 4.89, *P* = 0.030) reflecting a positive slope (0.007) in ex-arable fields (Fig 3). There was no effect of time for the chronosequence data (i.e., between-plot slope) (*F*_1,22_ = 1.87, *P =* 0.185) nor an interaction between chronosequence data and field type (*F*_1,22_ = 0.20, *P =* 0.661; Fig 3).

Non-native plant abundance did not change across the chronosequence in ex-arable or never-tilled fields (F_1,23_ = 0.00, *P* = 0.974; S1 Fig), but non-native abundance did decrease in ex-arable fields during direct observation (F_1,48_ = 6.49, *P* = 0.014) at a rate of -0.84% cover per year (S1 Fig). Native plant abundance did not change across the chronosequence in ex-arable or never-tilled fields (F_1,46_ = 0.75, *P* = 0.391), but native abundance decreased in never-tilled fields during direct observation (F_1,46_ = 7.2, *P* = 0.010) at a rate of -0.67% cover per year (S1 Fig).

Among native plant species, *A*. *tridentata* cover did not change during direct observation (F_1,106_ = 0.02, *P* = 0.895; S2 Fig) or the chronosequence (F_1,11_ = 4.07, *P* = 0.069). *B*. *sagittata* cover did not change during direct observation (F_1,86_ = 1.54, *P* = 0.218) or the chronosequence (F_1,11_ = 0.67, *P* = 0.432). *L*. *sericeus* cover increased in ex-arable fields during direct observation (F_1,69_ = 3.28, *P* = 0.002, slope = 0.23% per year) but not during the chronosequence (F_1,22_ = 0.00, *P* = 0.99). *P*. *spicata* cover decreased in never-tilled fields during direct observation (F_1,108_ = 7.59, *P* = 0.001, slope = -0.35% per year) but increased across the chronosequence (F_1,21_ = 2.66, *P* = 0.015, slope = 0.174). *P*. *tridentata* cover decreased during direct observation in never-tilled fields (F_1,48_ = 19.21, *P* < 0.001, slope = -0.42% per year), but not across the chronosequence (F_1,46_ = 0.17, *P* = 0.678).

Among non-native plants, *B*. *tectorum* cover decreased during direct observation in ex-arable fields (F_1,98_ = 5.97, *P* = 0.016; slope = -0.10; S2 Fig) and the chronosequence (F_1,22_ = 4.30, *P* = 0.050; slope = -0.004% per year). *C*. *diffusa* cover did not change during direct observation (F_1,124_ = 0.66, *P* = 0.509), but decreased across the chronosequence in ex-arable fields (F_2,30_ = 5.16, *P* = 0.012; slope = -0.13% per year). *C*. *draba* cover did not change during direct observation (F_1,48_ = 2.00, *P* = 0.164) or the chronosequence (F_1,23_ = 0.65, *P* = 0.43). *M*. *sativa* cover did not change across direct observation (F_1,48_ = 0.35, *P* = 0.558) or the chronosequence (F_1,23_ = 1.15, *P* = 0.295). *P*. *bulbosa* cover decreased during direct observation in both field types (F_1,48_ = 5.58, *P* = 0.022; slope = -0.277), but not across the chronosequence (F_1,46_ = 3.43, *P* = 0.070).

### Community composition responses to management, biocontrol and wildfire

**Fig 4:**
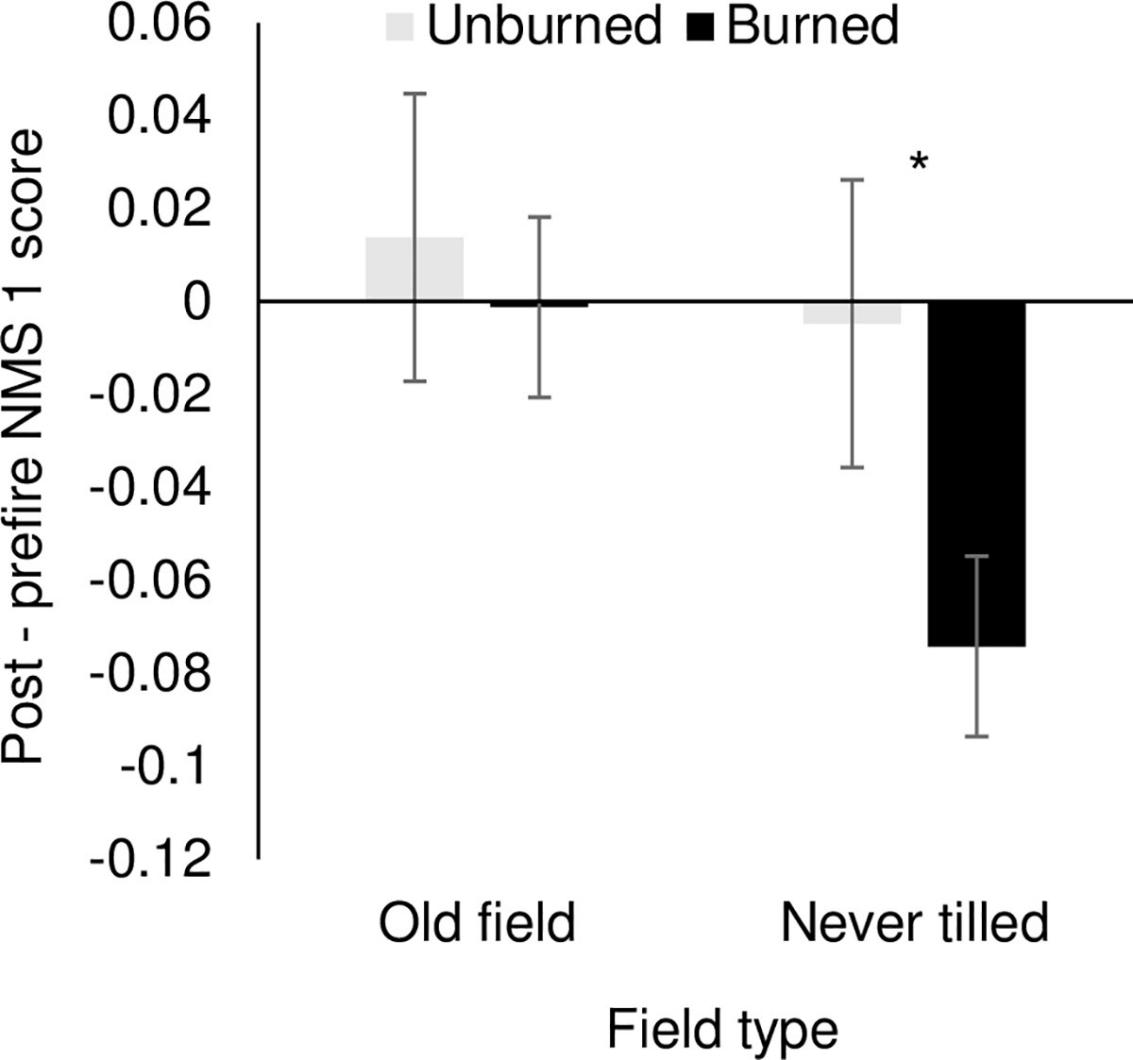
Change in NMS axis 1 values after a wildfire in 2014. Values calculated as the difference between 2015 scores and the average of 2002 to 2014 values. Negative post-prefire NMS scores indicate increased weediness. An asterisk indicates a difference between Unburned and Burned values at the α = 0.05 level.

When data from managed and unmanaged ex-arable fields were analyzed separately, communities in unmanaged fields became more similar to never-tilled fields while communities in managed fields did not show a directional change in composition [i.e., NMS axis 1 values increased with time in unmanaged fields (F_1,11_ = 18.38, *P* = 0.001), but not in managed fields (F_1,10_ = 2.07, *P* = 0.18; S3 Fig)]. In response to biocontrol treatment, cover of the target, *C*. *diffusa*, decreased to near zero in 2003 and 2004 and increased to become a dominant species in 2014 and 2015 (S2 Fig). In ex-arable fields, NMS axis 1 scores did not differ before and after wildfire (T_23_ = 0.43, *P* = 0.67, Fig 4). However, in never-tilled fields, NMS axis 1 values were lower after wildfire indicating that these fields became more like ex-arable fields (T_23_ = 3.90, *P* < 0.001, Fig 4).

## Discussion

Agricultural abandonment created two distinct plant communities that appear to function as alternative-state communities [12–13]. Fields that have been disturbed by agriculture and abandoned are dominated by short-lived non-natives. Never-tilled fields are dominated by long-lived natives. At the species and functional group levels, 13 years of direct observation revealed directional changes in both field types: non-native abundance decreased in ex-arable fields and native abundance decreased in never-tilled fields. These changes made the two communities more similar, but there was little evidence that the two community types would eventually converge because there was little species overlap between field types. Further, there was no evidence of directional changes in community composition across the chronosequence. In summary, the more-controlled, direct observation approach detected changes within communities over 13 years, but little species overlap and a lack of change in community composition across the chronosequence suggests that these communities are likely to remain distinct over long time periods (i.e., hundreds of years).

Communities in ex-arable and never-tilled fields are distinct (Fig 1). In ex-arable fields, the five most abundant species are non-native, short-lived (1 to 10 years) [37–42] and demonstrate wide fluctuations in percent cover from year to year (Fig 1 and S2 Fig). These ‘weedy’ communities have fewer species, less perennial cover, and more annual and forb cover than communities in never-tilled fields. In contrast, the five most abundant species in never-tilled fields are native, longer lived (>10 years) [37–42], and demonstrate more stable abundances over annual and decadal time scales (Fig 3).

While plant communities in ex-arable fields remained distinct from communities in never-tilled fields for 65 years, there was evidence of directional change in community composition in the 13 years of direct observation. Multivariate analyses suggested that plant communities in ex-arable fields were becoming more similar to plant communities in never-tilled fields (i.e., NMS 1 values increased in ex-arable fields during direct observation; Fig 3). This was consistent with a decline in the abundance of *B*. *tectorum* and *P*. *bulbosa*. Both species are small-statured, winter-active, non-native grasses that are dominant in ex-arable fields but uncommon in never-tilled fields. The only evidence that native species were becoming more abundant in ex-arable fields over time was for the nitrogen-fixing *L*. *sericeus*; total native cover in ex-arable fields did not change during direct observation or the chronosequence. Thus, while two common non-natives decreased and one native species increased, there was little evidence that ex-arable communities would return to native community composition over 50 to 100 year timescales. In contrast to ex-arable fields, there was no directional change in whole-community composition in never-tilled fields during direct observation. At the functional group and species-levels, however, there were declines in native plant abundance reflecting a decline in the common shrub, *P*. *tridentata*. There was no evidence that non-native plants were invading never-tilled fields. Broadly, direct observation data appeared sensitive to detecting within-community changes in species composition, but chronosequence data suggested that communities will remain dissimilar over long (60+ year) time periods.

There are several reasons why direct observation data may have revealed different patterns in community composition than chronosequence data [43]. First, unlike chronosequence data, direct observation data are not confounded by space-for-time substitutions. For example, differences in community composition among fields can ‘mask’ within-community changes over time in chronosequence datasets [10, 23–24]. Second, shorter-term, direct observation data are more likely to detect short-term linear patterns, even if longer-term patterns are non-linear. It is likely, for example, that weedy communities shift from annual to perennial dominance over five to ten year timescales, but that these communities then maintain dominance by perennial non-natives indefinitely. While the direct observation approach appeared more controlled and more sensitive to detecting community level changes, the inference it provides to community composition in the future is not as strong as from chronosequence data. It is possible, for example, that patterns observed in the direct observation dataset will be periodically ‘reset’ by disturbances from pocket gophers, drought, wildfire or human disturbance in ex-arable fields [10, 44–45]. Alternatively, it could be suggested that the direct observation data revealed a pattern in vegetation change that better reflects current and anticipated climate and management conditions (i.e., provides a better indicator of future change than the chronosequence data). While this may be occurring to some degree, the patterns observed during direct observation are unlikely to be maintained over longer time periods because 1) these directional changes were not observed in the chronosequence, and 2) plant cover was observed to decline in both field types during direct observation, but plant cover cannot be expected to decline indefinitely.

While late-successional species have often been observed to recolonize ex-arable fields [23, 46–47], in this system agricultural disturbance appears to have forced the community through a threshold to an alternative-state community dominated by non-native, early-successional plants [12–13]. Ex-arable fields were typically less than 200 m wide allowing native propagule pressure and results were similar in transects that were 5 m and 50 m from tillage boundaries (Fig 2). Ex-arable fields had less ground cover and more variable populations, which should have provided establishment opportunities for many generations of plants [10, 48]. Species in ex-arable fields likely experienced 30 to 60 generations during the chronosequence [37–42]. Even stands of the native shrubs, *A*. *tridentata* and *P*. *tridentata*, have been found to have mean ages less than 25 years in dendrochronological studies, and these species realize recruitment events every 2 to 3 years [25, 41–42, 49]. Thus, establishment, recruitment and mortality were expected in both the direct observation and chronosequence data in both communities [50], yet these communities remained distinct for 65 years.

Results stand in contrast to many studies demonstrating succession over similar time-scales [23–24, 46–47, 51–52]. It is not clear why succession would be rapid in some systems and not in others [53], though arid and semi-arid systems seem to be more likely to show very slow to no change relative to more mesic systems [6, 9, 19, 27, 52]. It is possible that slow succession or alternative-state communities are more likely in more stressful environments with greater facilitation [17, 53–57].

Direct observation data provided some insight into the processes through which short-lived non-natives may maintain dominance. It is particularly notable that the dominant species in ex-arable fields were resilient from changes in abundance. Perhaps the most dramatic example was that *C*. *diffusa* abundance declined to almost zero following the introduction of a biocontrol agent in 2003, then again became the dominant non-native species in 2014 and 2015. The temporary loss of *C*. *diffusa* was notable, but it was not the only source of variation in species abundances in ex-arable fields; most of the dominant species in ex-arable fields demonstrated large year-to-year changes in abundance. In addition to being resilient from changes in species-level abundances, ex-arable communities were resistant to changes caused by management and wildfire. Active and intensive management failed to shift ex-arable communities toward the composition of never-tilled fields and wildfire had no effect on ‘weediness’ in ex-arable fields (Fig 4). Together, these results illustrate that ex-arable plant communities were either resistant to or resilient from large disturbances (i.e., biocontrol addition, native plant restoration efforts or wildfire)[58–59]. In summary, results from both the chronosequence and direct observation are consistent with the idea that agricultural disturbance forces this ecosystem across a threshold to a new alternative state that is maintained by within-community feedbacks [12, 60–62].

## Supporting information

S1 Table Physical attributes of the 25 study fields in the Methow Valley, Washington, USA. Year = year abandoned from agricultural use. Managed = Year managed to restore native plant growth. Fire = Year of wildfire.
(DOCX)

S1 Fig Native (a and b) and non-native (c and d) ground cover in paired ex-arable (a and c) and never-tilled (b and d) fields. Each solid line represents plant-type abundance over 13 years of direct observation in a single field. Dotted lines represent significant best-fit regressions. Native plant cover in never-tilled fields (b) and non-native cover in ex-arable fields (c) decreased during 13 years of direct observation. There were no significant relationships between native or non-native cover and chronosequence time (i.e., 3 to 65 years).
(DOCX)

S2 Fig Ground cover of the five most common species in (A) ex-arable and (B) never-tilled fields over 13 years of direct observation. Each point represents mean cover in transects 50 m from tillage boundaries in 25 sites. The only correlation between ground cover and time observed was for PUTR = *Purshia tridentata*. MESA = *Medicago sativa*; BRTE = *Bromus tectorum*; CEDI = *Centaurea diffusa*; CADR = *Cardaria draba*; POBU = *Poa bulbosa*; PSSP = *Pseudoroegneria spicata*; BASA = *Balsamorhizae sagittata*; LUSE = *Lupinus sericeus*; ARTR = *Artemesia tridentata*.
(DOCX)

S3 Fig NMS axis 1 scores (species matrix) in old-field and never-tilled fields over time in the Chronosequence and Direct Observation datasets (A). Data from actively managed fields were separated from remaining data in the Direct Observation dataset (B). Data derived from plant species composition in 50-m transects. Positive y-axis values were associated with native; long-lived perennial communities (Fig 1). Since the chronosequence was observed for 13 years; each of the 25 fields sampled produced 13 age-since-abandonment values. Each data point in panel A therefore represents the average value for all fields measured at the indicated year (i.e.; year-since abandonment). In the Direct Observation dataset; a significant regression with time was observed in the unmanaged; old-field fields only. Note that half of the fields burned in a wildfire prior to the last data point.
(DOCX)
