# Supplementary material for: Chronosequence and direct observation approaches reveal complementary community dynamics in a novel ecosystem
Source: PLoS One. 2019 Mar 18;14(3):e0207047. doi: 10.1371/journal.pone.0207047 (PMC6422298; doi:10.1371/journal.pone.0207047)
Supplement: S1 Table — Year = year abandoned from agricultural use. Managed = Year managed to restore native plant growth. Fire = Year of wildfire. (DOCX) [file pone.0207047.s001.docx]

**S1 Table:** Physical attributes of the 25 study fields in the Methow Valley, Washington, USA**.** Year = year abandoned from agricultural use. Managed = Year managed to restore native plant growth. Fire = Year of wildfire.

| **#** | Site | Field size (ha) | Elevation  (m) | Aspect  (º) | Slope  (%) | Year | Managed | Fire |
| --- | --- | --- | --- | --- | --- | --- | --- | --- |
| 1 | Field 1* | 1.9 | 630 | 185 | 20 | 1950 | 2005,2006 | NA |
| 2 | Elbow 1 | 7.3 | 620 | 270 | 20 | 1999 | 2005,2006 | NA |
| 3 | Elbow 2 | 10.0 | 630 | 230 | 30 | 1996 | 2005,2006 | NA |
| 4 | Elbow N | 9.5 | 650 | 205 | 22 | 1996 | 2005,2006 | NA |
| 5 | Two Lakes | 2.3 | 700 | 355 | 15 | 1980 | 2009,2010 | NA |
| 6 | Moccasin | 1.7 | 770 | 355 | 18 | 1953 | NA | NA |
| 7 | Over Elbow | 3.2 | 810 | 150 | 15 | 1953 | NA | NA |
| 8 | Balky Hill | 16.4 | 780 | 200 | 14 | 1980 | 2010,2011 | 2014 |
| 9 | Fuzzy N | 4.4 | 830 | 205 | 15 | 1980 | 2010,2011 | 2014 |
| 10 | Fuzzy Middle | 7.8 | 780 | 54 | 20 | 1980 | 2010,2011 | 2014 |
| 11 | Fuzzy S | 4.8 | 780 | 180 | 19 | 1980 | 2010,2011 | 2014 |
| 12 | Pipestone N | 4.7 | 760 | 290 | 14 | 1970 | NA | 2014 |
| 13 | Pipestone Mid | 5.8 | 740 | 250 | 20 | 1970 | NA | 2014 |
| 14 | Pipestone S | 3.5 | 710 | 110 | 14 | 1993 | 2010,2011 | 2014 |
| 15 | Fuzzy Pipe | 4.0 | 700 | 290 | 18 | 1993 | 2010,2011 | 2014 |
| 16 | Hidden Weed | 8.3 | 880 | 205 | 23 | 1955 | NA | 2014 |
| 17 | Hidden Grass | 1.3 | 860 | 70 | 20 | 1955 | NA | 2014 |
| 18 | Lower Lester | 1.3 | 990 | 20 | 18 | 1992 | NA | 2014 |
| 19 | Upper Lester | 2.8 | 1000 | 170 | 20 | 1992 | NA | 2014 |
| 20 | Campbell | 2.5 | 880 | 40 | 16 | 1980 | NA | 2014 |
| 21 | Cougar N | 6.3 | 950 | 210 | 11 | 1996 | NA | 2014 |
| 22 | Cougar S | 3.1 | 950 | 200 | 29 | 1973 | NA | 2014 |
| 23 | Half disked | 9.6 | 840 | 210 | 23 | 1990 | NA | NA |
| 24 | Lloyd ranch | 9.5 | 680 | 206 | 21 | 1998 | 2010,2011 | NA |
| 25 | Haas | 16.7 | 850 | 210 | 22 | 1955 | NA | NA |

**Latitude and longitude of each site provided in Kulmatiski (2006)*
