# Supplementary material for: Chronosequence and direct observation approaches reveal complementary community dynamics in a novel ecosystem
Source: PLoS One. 2019 Mar 18;14(3):e0207047. doi: 10.1371/journal.pone.0207047 (PMC6422298; doi:10.1371/journal.pone.0207047)
Supplement: S1 Fig — Native (a and b) and non-native (c and d) ground cover in paired ex-arable (a and c) and never-tilled (b and d) fields. Each solid line represents plant-type abundance over 13 years of direct observation in a single field. Dotted lines represent significant best-fit regressions. Native plant cover in never-tilled fields (b) and non-native cover in ex-arable fields (c) decreased during 13 years of direct observation. There were no significant relationships between native or non-native cover and chronosequence time (i.e., 3 to 65 years). (DOCX) [file pone.0207047.s002.docx]

**S1 Fig**. Native (a and b) and non-native (c and d) ground cover in paired ex-arable (a and c) and never-tilled (b and d) fields. Each solid line represents plant-type abundance over 13 years of direct observation in a single field. Dotted lines represent significant best-fit regressions. Native plant cover in never-tilled fields (b) and non-native cover in ex-arable fields (c) decreased during 13 years of direct observation. There were no significant relationships between native or non-native cover and chronosequence time (i.e., 3 to 65 years).
