# Supplementary material for: Chronosequence and direct observation approaches reveal complementary community dynamics in a novel ecosystem
Source: PLoS One. 2019 Mar 18;14(3):e0207047. doi: 10.1371/journal.pone.0207047 (PMC6422298; doi:10.1371/journal.pone.0207047)
Supplement: S2 Fig — Ground cover of the five most common species in (A) ex-arable and (B) never-tilled fields over 13 years of direct observation. Each point represents mean cover in transects 50 m from tillage boundaries in 25 sites. The only correlation between ground cover and time observed was for PUTR = Purshia tridentata. MESA = Medicago sativa; BRTE = Bromus tectorum; CEDI = Centaurea diffusa; CADR = Cardaria draba; POBU = Poa bulbosa; PSSP = Pseudoroegneria spicata; BASA = Balsamorhizae sagittata; LUSE = Lupinus sericeus; ARTR = Artemesia tridentata. (DOCX) [file pone.0207047.s003.docx]

**S2 Fig**. Ground cover of the five most common species in (A) ex-arable and (B) never-tilled fields over 13 years of direct observation. Each point represents mean cover in transects 50 m from tillage boundaries in 25 sites. The only correlation between ground cover and time observed was for PUTR *= Purshia tridentata*. MESA = *Medicago sativa;* BRTE *= Bromus tectorum;* CEDI *= Centaurea diffusa;* CADR *= Cardaria draba;* POBU *= Poa bulbosa;* PSSP *= Pseudoroegneria spicata;* BASA *= Balsamorhizae sagittata;* LUSE *= Lupinus sericeus;* ARTR *= Artemesia tridentata.*
