# Supplementary material for: Chronosequence and direct observation approaches reveal complementary community dynamics in a novel ecosystem
Source: PLoS One. 2019 Mar 18;14(3):e0207047. doi: 10.1371/journal.pone.0207047 (PMC6422298; doi:10.1371/journal.pone.0207047)
Supplement: S3 Fig — NMS axis 1 scores (species matrix) in old-field and never-tilled fields over time in the Chronosequence and Direct Observation datasets (A). Data from actively managed fields were separated from remaining data in the Direct Observation dataset (B). Data derived from plant species composition in 50-m transects. Positive y-axis values were associated with native; long-lived perennial communities (Fig 1). Since the chronosequence was observed for 13 years; each of the 25 fields sampled produced 13 age-since-abandonment values. Each data point in panel A therefore represents the average value for all fields measured at the indicated year (i.e.; year-since abandonment). In the Direct Observation dataset; a significant regression with time was observed in the unmanaged; old-field fields only. Note that half of the fields burned in a wildfire prior to the last data point. (DOCX) [file pone.0207047.s004.docx]

**S3 Fig**. NMS axis 1 scores (species matrix) in ex-arable fields and never-tilled fields over time in the Chronosequence and Direct Observation datasets (A). Data from actively managed fields were separated from remaining data in the Direct Observation dataset (B). Data derived from plant species composition in 50-m transects. Positive y-axis values were associated with native; long-lived perennial communities (Fig 1). Since the chronosequence was observed for 13 years; each of the 25 fields sampled produced 13 age-since-abandonment values. Each data point in panel A therefore represents the average value for all fields measured at the indicated year (i.e.; year-since abandonment). In the Direct Observation dataset; a significant regression with time was observed in the unmanaged; ex-arable fields only. Note that half of the fields burned in a wildfire prior to the last data point.
